# Noninvasive intracranial pressure monitoring methods: a critical review

**DOI:** 10.1590/0004-282X-ANP-2020-0300

**Published:** 2021-05-01

**Authors:** Fabiano Moulin de Moraes, Gisele Sampaio Silva

**Affiliations:** 1 Universidade Federal de São Paulo Departamento de Neurologia e Neurocirurgia Unidade Neurovascular São Paulo SP Brazil Universidade Federal de São Paulo, Departamento de Neurologia e Neurocirurgia, Unidade Neurovascular, São Paulo SP, Brazil.

**Keywords:** Brain Injury, Intracranial Hypertension, Stroke, Intracranial Pressure, Head Trauma, Lesão Encefálica, Hipertensão Intracraniana, Acidente Vascular Cerebral, Pressão Intracraniana, Trauma Craniano

## Abstract

**Background:**

Intracranial pressure (ICP) monitoring has been used for decades in management of various neurological conditions. The gold standard for measuring ICP is a ventricular catheter connected to an external strain gauge, which is an invasive system associated with a number of complications. Despite its limitations, no noninvasive ICP monitoring (niICP) method fulfilling the technical requirements for replacing invasive techniques has yet been developed, not even in cases requiring only ICP monitoring without cerebrospinal fluid (CSF) drainage.

**Objectives:**

Here, we review the current methods for niICP monitoring.

**Methods:**

The different methods and approaches were grouped according to the mechanism used for detecting elevated ICP or its associated consequences.

**Results:**

The main approaches reviewed here were: physical examination, brain imaging (magnetic resonance imaging, computed tomography), indirect ICP estimation techniques (fundoscopy, tympanic membrane displacement, skull elasticity, optic nerve sheath ultrasound), cerebral blood flow evaluation (transcranial Doppler, ophthalmic artery Doppler), metabolic changes measurements (near-infrared spectroscopy) and neurophysiological studies (electroencephalogram, visual evoked potential, otoacoustic emissions).

**Conclusion:**

In terms of accuracy, reliability and therapeutic options, intraventricular catheter systems still remain the gold standard method. However, with advances in technology, noninvasive monitoring methods have become more relevant. Further evidence is needed before noninvasive methods for ICP monitoring or estimation become a more widespread alternative to invasive techniques.

## RESUMO

**Introdução:**

O uso da monitorização da pressão intracraniana (PIC, em sua sigla em inglês) é adotado há décadas no manejo de diversas condições neurológicas. O padrão ouro atual é a monitorização invasiva intraventricular, que está relacionada a inúmeras complicações. Apesar dessas limitações, até o momento nenhum método de monitorização não invasiva (niPIC, em sua sigla em inglês) conseguiu substituir a técnica invasiva.

**Objetivos:**

Revisar os métodos não invasivos de monitorização da PIC.

**Métodos:**

As diferentes modalidades e abordagens foram agrupadas de acordo com o mecanismo utilizado para detectar elevação da PIC ou suas consequências.

**Resultados:**

As técnicas descritas foram: o exame físico, neuroimagem (tomografia computadorizada e ressonância magnética de crânio), estimativas indiretas da PIC (fundoscopia, deslocamento da membrana timpânica, elasticidade craniana), avaliação do fluxo cerebral (doppler transcraniano e doppler da artéria oftálmica), alterações metabólicas (Espectroscopia próxima do infravermelho) e estudos neurofisiológicos (eletroencefalograma, potencial evocado visual e emissões otoacústicas).

**Conclusão:**

Considerando a acurácia, confiabilidade e opções terapêuticas, o sistema de cateteres intraventricular ainda permanece como padrão ouro. No entanto, com os avanços tecnológicos, os métodos não invasivos têm se tornados mais relevantes. Mais evidências são necessárias antes que essas modalidades de monitorização ou estimativas não invasivas se tornem uma alternativa mais robusta às técnicas invasivas.

## INTRODUCTION

Intracranial pressure (ICP) monitoring has been used for decades in management of various neurological conditions (Table 1) and has become a staple of neurocritical care^1^. While management of ICP is of clear benefit, there is no consensus in the literature about whether ICP monitoring provides any clinical benefit, compared with management based only on the patient's neurological examination, imaging findings and the clinician's judgment^2^^,^^3^. While some studies have shown that ICP monitoring is associated with improved survival rates, others have suggested that this not only is fruitless but also may, in fact, lead to worse clinical outcomes, including increased mortality, longer hospitalization, increased complication rates and increased hospitalization costs, compared with a non-ICP monitoring approach in patients with traumatic brain injury (TBI)^4^. The only randomized trial assessing the effect of invasive ICP monitoring on clinical outcomes, conducted by Chesnut et al.^5^ among patients with severe TBI, found that there was no significant difference in six-month mortality. These results indicate that there is still room for improvement of clinical management of ICP monitoring findings, which could assist in better clinical decisions and improved outcomes for critically ill patients^2^^,^^4^.

**Table 1:** Potential indications for intracranial pressure monitoring.

Traumatic brain injury
Intracranial hemorrhage
Subarachnoid hemorrhage
Cerebral edema
Hydrocephalus
Hepatic encephalopathy
Cerebral ischemia

A ventricular catheter connected to an external strain gauge is the gold standard for measuring ICP. This is an invasive system associated with a number of complications, including hemorrhage, obstruction, mispositioning, infection and loss of accuracy for asymmetric hemispheric lesions, besides requiring a neurosurgical procedure^6^^,^^7^. However, despite its limitations, no noninvasive ICP monitoring (niICP) method fulfilling the technical requirements for replacing invasive techniques has yet been developed, not even in cases requiring only ICP monitoring without cerebrospinal fluid (CSF) drainage^8^^,^^9^.

Here, we review the current methods for monitoring niICP and the neurological consequences of increased ICP, such as reduced cerebral blood flow (CBF) and metabolic changes^10^. The different methods and approaches were grouped according to the mechanism used for detecting elevated ICP or its associated consequences. The main approaches reviewed here were: physical examination, brain imaging (magnetic resonance imaging [MRI], computed tomography [CT] and optic nerve sheath ultrasound [ONS-US]), indirect ICP estimation techniques (fundoscopy, tympanic membrane displacement and skull elasticity), cerebral blood flow velocity (transcranial Doppler [TCD] and ophthalmic artery Doppler), metabolic changes measurements (near-infrared spectroscopy [NIRS]) and neurophysiological studies (electroencephalogram [EEG], visual evoked potential [VEP] and otoacoustic emissions) (Table 2). Although this topic has been addressed by other authors before, we present an updated review of the literature with a discussion of new methods not previously evaluated in narrative reviews.

**Table 2:** Noninvasive indications for intracranial pressure monitoring methods.

Methods studied
***Physical examination***
***Neuroimaging***
CT and MRI of the brain
US of the optic nerve sheath
***Metabolic changes*** Near-infrared spectroscopy
***Indirect ICP monitoring***
Skull elasticity
Anterior fontanelle pressure
Venous ophthalmodynamometry
Acoustic elasticity
Pupillometry
Tympanic membrane displacement
***Metabolic changes***
Near-infrared spectroscopy
***Neurophysiology***
Electroencephalogram
Visual evoked potential
Otoacoustic emissions
***Cerebral blood flow***
Transcranial Doppler

ICP: indications for intracranial pressure.

### Physical examination

Because patients requiring ICP monitoring usually have severe and acute neurological conditions, neurological parameters in such patients are often derived from the Glasgow Coma Scale (GCS), which is the most common scoring system used in these settings. Only three physical examination findings, which had been evaluated in an adequate number of relevant studies and were included in a meta-analysis, correlated with increased ICP: pupillary dilation; motor posturing, defined by GCS motor score ≤3; and decreased level of consciousness, defined by total GCS≤8. The presence of pupillary dilation had a sensitivity of 28.2% and specificity of 85.9% for the diagnosis of elevated ICP, whereas the presence of motor posturing had a sensitivity of 54.3% and specificity of 63.6%. Lastly, a decreased level of consciousness had a sensitivity of 75.8% and specificity of 39.9% for the diagnosis of elevated ICP^1^.

### Neuroimaging

#### Computed tomography and magnetic resonance imaging of the brain

**Figure 1:**
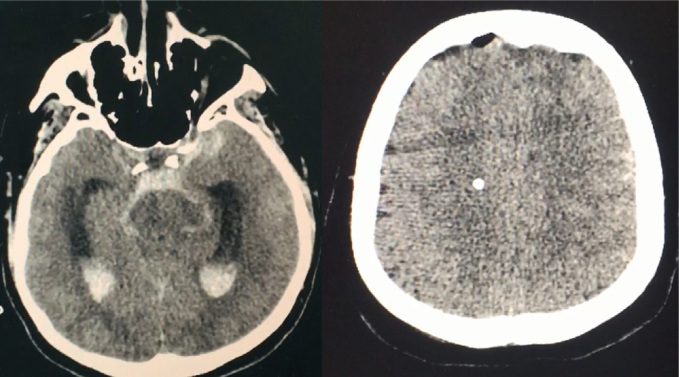
Computed tomography findings in intracranial hypertension (A) loss of cortical-subcortical differentiation and cortical subarachnoid hemorrhage. (B) temporal horn dilation and cisternal subarachnoid hemorrhage.

CT and MRI of the brain are routinely used for diagnosing neurological disorders and can provide qualitative information about ICP. A variety of gross anatomical changes associated with elevated ICP can be detected using brain imaging techniques, including lateral ventricle compression, midline shift, ventricular dilation and loss of cortical-subcortical differentiation^11^^,^^12^(Figures 1A and 1B). Most CT-based studies have been conducted on patients with TBI, and the Marshall classification is the most commonly used classification of head injury, based primarily on CT findings^13^ (Table 3). However, it is worth emphasizing that most brain injury rating scales were designed for prognostic purposes and not necessarily for ICP monitoring. The presence of other CT and MRI signs in the setting of brain injury, suggestive of raised ICP, has also been used to guide the management of elevated ICP (Table 4)^14^.

**Table 3:** Marshall scale.

Type	Types of abnormalities on CT scanning
I	No visible pathological condition on CT scan
II	Cisterns present with midline shift 0‒5 mm; no lesion >25 mL
III	Cisterns compressed or absent with midline shift 0‒5 mm; no lesion >25 mL
IV	Midline shift >5 mm; no lesion >25 mL
V	Any lesion surgically evacuated
VI	Lesion >25 mL not surgically evacuated

CT: computed tomography; MRI: magnetic resonance imaging; CT: computed tomography.

**Table 4:** Computed tomography/magnetic resonance imaging findings suggestive of elevated indications for intracranial pressure.

1) Diffuse sulcal effacement
2) Effacement of basal cisterns
3) Hydrocephalus defined as: Both temporal horns >2 mm Evans’ index*
4) Midline shift >5 mm
5) Transtentorial or uncal herniation

* The Evans’ index is the ratio of maximum width of the frontal horns of the lateral ventricles, divided by the maximum internal diameter of the skull at the same level, used in axial computed tomography and magnetic resonance imaging images.

A normal brain CT at admission among patients with TBI does not rule out the risk of either early intracranial hypertension or possible development of elevated ICP, with predictive values ranging from 0 to 88%^15^. A small study demonstrated that it was possible to differentiate between normal and elevated ICP using a CT-determined ratio of CSF volume to total intracranial volume, with a predictive accuracy of 67%^8^^,^^11^. However, CT-based criteria have high specificity but low sensitivity, and thus high false-negative rates^11^.

MRI is more sensitive but rarely available and more time consuming than CT. Thus, it is not widely applied for ICP monitoring. In a small pilot study, an MRI-based technique for estimating ICP by assessing net transcranial blood and CSF flow was able to differentiate between patients with normal or elevated ICP^12^. An elastance index was derived from the ratio of intracranial pressure to volume change. Briefly, pulsatile arterial, venous and CSF flow in and out of the cranial vault during the cardiac cycle causes a small volume change that was measured. The elastance index correlated extremely well with the invasively measured ICP (r^2^=0.965; p<0.005). However, care is required in selecting representative images on slides and a representative blood vessel, and the technique offers only a picture of ICP within a particular time frame^12^.

In short, even though neuroimaging techniques continue to be used qualitatively, these methods are currently not sufficiently reliable as monitoring tools for elevated ICP^16^.

#### Optic nerve sheath diameter

At the point at which the optic nerve exits the intracranial space into the orbit, it is still surrounded by the dural sheath. As such, the subarachnoid space (SAS) surrounding the nerve is contiguous with the intracranial subarachnoid space. Elevation of ICP can transmit through the CSF in the subarachnoid space, leading to dilation of the optic nerve sheath (ONS), which can be detected using transocular ultrasonography^14^^,^^17^.

Several studies have demonstrated a correlation between invasively measured ICP and ultrasonographic ONS diameter (ONSD) measurements, with overall sensitivity and specificity of 0.95 and 0.92 for detecting elevated ICP, depending on the cutoff for detection of raised ICP, which ranges from 4.8 mm^14^ to 5.6 mm^18^. A few reasons for such variations have been put forward. One is that the there are two different measurement techniques: coronal and axial techniques, with different accuracies. The coronal technique presents less variability, but the axial method provides a better estimate of ICP. A second reason is that studies have either averaged measurements between eyes or chosen to evaluate the highest measurement between the eyes, which naturally creates the discrepancies found in the literature^18^.

**Figure 2:**
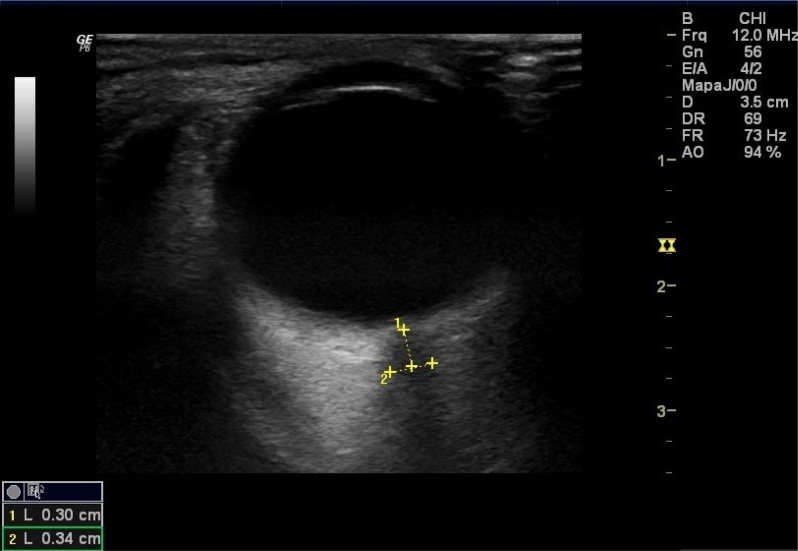
Optic nerve sheath and the globe are evident. The optic nerve sheath is a linear hypoechoic structure posterior to the globe. Line 1 identifies the site of optic nerve sheath diameter measurement 0.3 cm behind the retina. Line 2 measures the optic nerve sheath diameter (0.34 cm in this case).

At present, this variation in the optimal ONSD cutoff makes a formal meta-analysis approach impractical^16^. The ONSD is measured at a depth of 3 mm from the posterior pole of the globe, as this point is most reflective of the changes in ICP (Figure 2). While intra and interobserver variability seem to be lower than that of transcranial Doppler (TCD), this method cannot be used in patients with face trauma or lesions of the orbit such as Grave's disease and sarcoidosis. Additionally, there is some evidence that the specificity of ONSD declines when there are acute fluctuations in ICP^17^. Nevertheless, ONSD measurements seem to be useful as a screening test for ICP in settings where invasive monitoring is not promptly available. Other ophthalmological approaches such as optical coherence tomography (OCT) have also been evaluated for ICP measurement^19^. The ONSD can also be measured using CT and MRI, but the accuracy of measurements by these two methods is lower^20^.

### Cerebral blood flow evaluation

#### Transcranial Doppler

**Figure 3:**
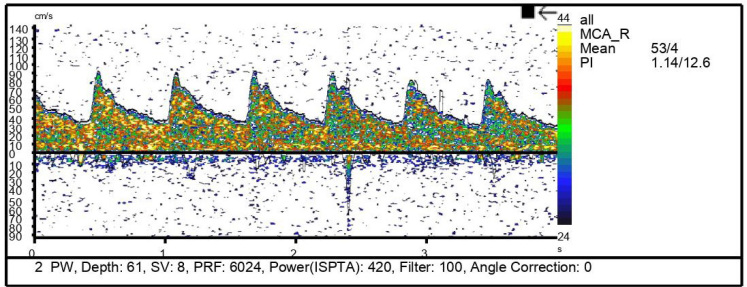
Transcranial Doppler measurement of right middle cerebral artery flows, demonstrating a PI of 1.26.

In the neurocritical setting, transcranial Doppler (TCD) is most commonly used as a tool for monitoring changes in cerebral blood flow (CBF) velocity in the setting of subarachnoid hemorrhage (SAH) and its complications, including vasospasm^21^. A number of models using TCD-derived parameters have been used to assess correlations with invasively measured ICP^22^. These models have used measurements of flow velocity (FV) in the middle cerebral artery (MCA), arterial blood pressure and pulsatility index (PI)^23^. PI is derived from the TCD waveform and is defined as the difference between systolic and diastolic flow velocities, divided by the mean FV. Wakerley et al.^24^ developed a formula to predict ICP using PI − ICP = 10.93 × PI − 1.28 (Figure 3). Some studies have shown good correlation between ICP and PI values in patients with TBI, with sensitivity and specificity of 0.89 and 0.92, respectively, for detecting elevated ICP^23^.

Other studies have reported more modest results. Zweifel et al.^25^ prospectively analyzed a cohort of 290 patients with TBI and found a poor correlation between PI and ICP (0.31; p=0.001). They concluded that the value of PI for assessing ICP was limited. On the other hand, Schmidt et al.^26^ applied a black-box mathematical model for niICP assessment with good results. A recent prospective study found that using a model that combined all TCD-derived data was superior to a model in which these data were accessed individually for estimating ICP, with a correlation coefficient of r=0.47 (p<0.05) and an area under the curve of 0.73 (p<0.05). However, TCD has some limitations: it requires training and repetitive exercise; there is intra and interobserver variability; it is not useful for patients requiring continuous monitoring; and it cannot be used on 10–15% of the patients due to a lack of bone window^27^.

Other methods using Doppler technology include venous transcranial Doppler^28^ and ophthalmic artery Doppler^29^, but few studies have been conducted and both of these techniques have technical difficulties that prevent their widespread adoption into clinical care.

### Metabolic changes

#### Near-infrared spectroscopy

Near-infrared spectroscopy (NIRS) is a noninvasive technology that has been under development for assessment of ICP-related alterations in cerebral oxygenation^30^. NIRS sensors emit near-infrared (NIR) light onto the surface of the head and work on the principle of differential absorption of light in the vicinity of the infrared spectrum to detect changes in oxygen and hemoglobin concentration. Changes to the underlying tissue characteristics affect light absorption and diffusion, and spectral analysis can be used to garner information about tissue state and estimated intracranial oxygen saturation, which consequently reflects brain metabolism. This can also be used to detect changes in brain tissue oxygenation, cerebral blood volume and cerebral blood flow^30^.

Even though NIRS has been successfully used for monitoring oxygenation in various procedures, its reduced accuracy as a result of the effects of scalp and skull injury, as well as possible pathological changes in baseline saturation, has made this technology unreliable for widespread use^31^. Additionally, it cannot be used to estimate absolute ICP but, rather, changes in cerebral perfusion pressure (CPP) and brain oxygenation. Lastly, most brain injuries are heterogeneous and the technology does not take these variations into account.

### Neurophysiology

#### Electroencephalogram

Numerous studies have explored the concept that neurophysiological changes may actually precede ICP changes in patients with intracranial hypertension^32^^,^^33^. Because changes in ICP affect cerebral perfusion, neuronal activity and brain metabolism, some EEG patterns may be useful for ICP monitoring^32^^,^^33^. Specific EEG tracings correlate closely with changes in cerebral blood flow (CBF). In particular, changes in ICP correlate significantly with EEG burst duration^34^. Other studies have investigated more complex technologies such as power spectrum analysis^35^, entropy and bispectral index (BIS) and their correlations with CPP and ICP, but no significant applicability within clinical practice was found^36^.

#### Visual evoked potential

York et al.^37^ demonstrated a good relationship between ICP elevation and a shift in latency of the N2 wave of the visual evoked response. The N2 wave is normally found at 70 ms and corresponds to a cortical phenomenon. It is therefore likely to be sensitive to cerebral cortical injuries and increased ICP. Despite some positive results, Andersson et al.^38^ demonstrated that VEP has a wide range of latencies, amplitudes and waveforms across normal subjects. In addition, a large proportion of their subjects also had high intra-individual variability over time, which made VEP unreliable as a marker for ICP.

### Indirect intracranial pressure estimation

#### Pupillometry

In 1983, Marshall et al. established the fact that the oval pupil represented a stage between the normal pupil and the fixed unreactive pupil of patients with high ICP, and concluded that an oval pupil was indicative of high ICP^39^. However, their study did not indicate any specific numerical values of ICP correlating with pupillary shape changes. Since then, many studies have been conducted to assess pupillary changes in severely ill patients, to assess their outcome and clinical management. More recently, Chen et al*.* introduced the neurological pupillary index (NPI) as an early indicator of increased ICP^40^. Quantitative assessment of pupillary reactivity measured using a pupillometer showed excellent accuracy in comparison with invasively measured ICP. However, there still is no direct correlation between NPI and real values of ICP, and pupillometers cannot be used to continuously monitor ICP in intensive care units (ICUs)^40^.

#### Skull elasticity

The Monro–Kellie doctrine states that after closure of the fontanelles, the volume inside the cranium remains constant at all times, and that there is no deformation of the skull caused by changes in ICP^41^^,^^42^^,^^43^. However, questions arising from this assertion have recently created a new avenue of study in the field of ICP monitoring. The notion of measuring minute expansions of the skull as a reflection of increasing ICP was first explored in dogs and cadavers by Pitlyk et al.^44^ in 1985, but the technology that they used did not allow them to go any further. In 2009, Yue and Wang confirmed the previous findings and showed that there was a positive correlation between increasing ICP and skull deformation in rats, with excellent instrument sensitivity^45^.

**Figure 4:**
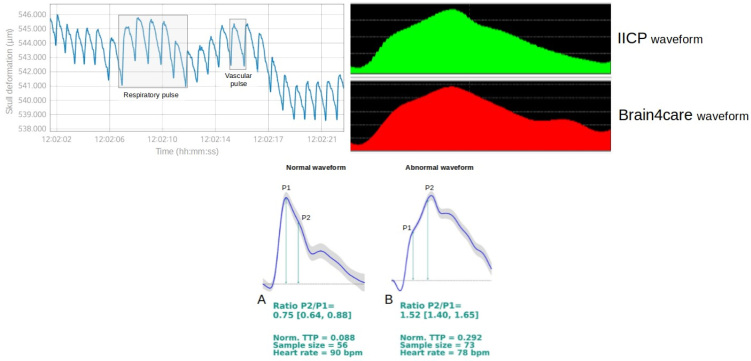
Waveform analysis. (A) Components of the indications for intracranial pressure waveform: pulse pressure waveform and respiratory waveform. (B) Comparison of indications for intracranial pressure waveforms obtained with an invasive sensor (iICP) and the noninvasive Brain4care® system. (C) indications for intracranial pressure waveforms measured using the Brain4care® sensor. A: normal waveform (P1>P2). B: abnormal waveform (P2>P1). The report provides quantitative information about ICP wave morphology to assist in patient assessment and follow-up. P2/P1 ratio: the ratio between the amplitudes of peaks P2 and P1; TTP: time to peak, defined as the time, from the start of the pulse, at which the ICP waveform reaches its highest peak.

More recently, a novel noninvasive technology (Brain4care®) was developed to detect very small variations in the volume of the skull caused by changes in ICP, without the need for surgery or even for the patient's head to be shaved. In this device, a strain sensor is placed in contact with the skin surface at the temporoparietal transition, lateral to the sagittal suture^46^^,^^47^. Non-invasive contact with the skull is achieved by applying adequate pressure directly on the scalp, using a pin, which requires minimal training. At the current stage of development, the device does not display calibrated pressure values in mmHg, but it can deliver continuous, real-time information about the ICP waveform and, consequently, brain compliance. The information shows great similarity to the curves obtained using invasive methods (Figures 4A and 4B)^46^^,^^47^^,^^48^.

#### Intracranial pressure waveforms and skull elasticity

In order to better understand this technology, it is important to note that mean ICP is a time-average of the ICP waveform. The ICP waveform consists of three components: respiratory waveforms (0.1‒0.3 Hz) associated with the respiratory cycle, pulse pressure waveforms of frequency equal to the heart rate and slow vasogenic waveforms (e.g. Lundberg A and B waves)^2^^,^^3^ (Figure 4A). The pulse pressure waveform is subdivided into three waves: P1 (percussion wave), which represent arterial pulsation transmitted from the choroid plexus; P2 (tidal wave), which reflect rebound pulsations of the brain parenchyma and are a proxy for intracranial compliance; and P3 (dicrotic wave), which represent pressure transmission as a result of aortic valve closure (Figure 4C). Elevated ICP also affects the characteristics of the ICP waveform. For example, an increase in the amplitudes of the three peaks indicates an increase in mean ICP; a reduction in the P1 amplitude suggests decreased cerebral perfusion; and an increase in P2 indicates loss of brain compliance (Figure 4C). Fusion of the peaks P1, P2 and P3 in association with high mean amplitude is an indicator of loss of cerebrovascular autoregulation and loss of cerebral perfusion. In addition, the presence of Lundberg A waves, which are sustained increases in mean ICP lasting 5‒20 min, may also signify diminished compliance. Lundberg B waves, which are clustered cyclic elevations in ICP occurring at a rate of 0.33‒3 cycles per min with overall cluster duration of 5‒30 min, are non-specific indicators of elevated ICP, given that they can also be present in patients with normal ICP^2^^,^^3^.

It is important to emphasize that in this novel technology, monitoring responds promptly to variations in ICP, with either increases or decreases, without delay or rebounds, thus confirming that no bone hysteresis occurs^49^ (i.e. the tendency of a system to preserve a deformation effected by a stimulus). Thus, micrometric deformations of the skull bones that are caused by and correlate linearly with changes in ICP can be detected. Measurements of ICP waveforms using this technology have been made in animal models and in human adults and children^50^^,^^51^^,^^52^.

## OTHER METHODS

Venous ophthalmodynamometry, which is based on the idea of measuring central retinal vein (CRV) pressure as a surrogate of ICP, was first proposed in 1925^53^. However, it was only at the end of the last century that the idea was explored in a study^54^. It was concluded that CRV pressure measurement or ophthalmodynamometry showed good correlation with invasive ICP monitoring. However, this technology is not useful for continuous monitoring. Subsequent refinements to the technology have improved its accuracy (sensitivity of 84.2% and specificity of 92.8%) for predicting raised ICP^55^.

The concept of using tympanic membrane displacement (TMD) as a surrogate for ICP is based on the proximity of the stapes and the oval window. The assumption is that the cochlear fluid pressure, which would be a function of the ICP, could affect the stapedial excursions^56^. The evidence available has shown that TMD is a good screening tool that can be useful in assessment and follow-up of patients at risk of increased ICP. However, TMD does not allow establishment of specific ICP values, is not useful for continuous monitoring and requires intact auditory anatomy^57^^,^^58^. Another technique that uses the ear as a surrogate for ICP is distortion-product otoacoustic emissions (DPOAE)^59^. Studies correlating DPOAE with ICP in experimental models and humans have shown good correlation with invasive methods. DPOAE measurements can possibly be an effective tool in non-invasive monitoring of ICP. However, the technique has some limitations: it does not allow absolute measurement of ICP; there is substantial inter-individual variability; and it can only be used in normal-hearing subjects^60^^,^^61^.

Tissue resonance analysis^62^ and acoustoelasticity^63^ use the tissue-specific ultrasound resonance of the brain and have shown good correlation with ICP in experimental models. However, there is a scarcity of studies on humans and the level of evidence remains poor.

In infants, the anterior fontanelle is open, and anterior fontanelle pressure monitoring presents a window for measuring ICP noninvasively^64^^,^^65^. However, despite multiple studies and various devices used to measure anterior fontanelle pressure, ICP monitoring in infants is currently not feasible in clinical practice through non-invasive methods^66^. Similarly, attempts to correlate intraocular pressure (IOP) with ICP using ocular tonometry, despite its potential, are not supported by the current evidence as a form of noninvasive ICP monitoring^67^^,^^68^^,^^69^.

## DISCUSSION

The search for a completely noninvasive intracranial pressure (niICP) technique capable of real-time monitoring is the Holy Grail of neurocritical care practice and research^1^^,^^3^. If available, it would have a wide range of applications in neurosurgery, neurosciences and translational medicine, from exercise physiology to aerospace medicine.

Despite recent advances that have led to development of various noninvasive techniques for monitoring ICP, the current noninvasive techniques cannot be used as an alternative to the invasive ones^70^. Ideally, a niICP monitoring technique should have the following attributes: to be simple and convenient to use; to depend little on operator experience and bone window; to be readily available throughout the hospital; to provide continuous monitoring; to be quantitative rather than qualitative; track dynamic changes in ICP and CPP; to be less influenced by the patient's cardiovascular instability; and, obviously, to be accurate. All noninvasive techniques have their own advantages and disadvantages, but no method currently satisfies all the criteria for replacing invasive ICP monitoring (Table 5).

**Table 5:** Comparison of the main noninvasive indications for intracranial pressure monitoring methods.

	CT/MRI	TCD	ONSD	NIRS	Pupillometry	Skull elasticity	EEG
Portability	No	Yes	Yes	Yes	Yes	Yes	Yes
Operator experience	No	Yes	Yes	No	No	No	Yes
Continuous monitoring	No	No	No	No	No	Yes	Yes
Cost per patient	Moderate	low	low	low	low	low	low
Complications	Yes	No	No	No	No	No	No

CT: computed tomography; MRI: magnetic resonance imaging; TCD: transcranial doppler; ONSD: optic nerve sheath diameter; NIRS: near-infrared spectroscopy; EEG: electroencephalogram.

Promising prospects include continuously combining noninvasive methods with other clinical or invasive parameters and integrating the data to improve pathophysiological understanding in suspected or confirmed cases of intracranial hypertension. This would help in identifying which types of monitoring can ideally be combined (e.g. oxygenation + compliance+ hemodynamics) and in which clinical setting they can contribute to the care and outcome of neurocritical patients (initial screening and continuous or intermittent monitoring). One limitation of this and other reviews of the topic is the lack of systematic evaluation of the data. In order to perform a systematic review and calculate accuracy between methods, good-quality data comparing all the methods with the gold standard (invasive intracranial pressure monitoring) would be needed. Unfortunately, such data are not available in the current literature^2^^,^^8^^,^^16^. The challenge of continuous and accurate niICP monitoring remains daunting, but the reward for patients and for science as a whole makes every effort sensible and commendable.

ICP monitoring has become established as a useful method for predicting outcomes and guiding therapy for patients suffering from a range of neurological conditions. In terms of accuracy, reliability and therapeutic options, intraventricular catheter systems still remain the gold standard method. However, with advances in technology, noninvasive monitoring methods have become more relevant. Further evidence is needed before noninvasive ICP monitoring can become a more widespread alternative to invasive techniques.
